# Fatty Liver Index Independently Predicts All-Cause Mortality in Patients With Antineutrophil Cytoplasmic Antibody-Associated Vasculitis but No Substantial Liver Disease

**DOI:** 10.3389/fcvm.2022.848121

**Published:** 2022-06-23

**Authors:** Pil Gyu Park, Jung Yoon Pyo, Sung Soo Ahn, Hyun Joon Choi, Jason Jungsik Song, Yong-Beom Park, Ji Hye Huh, Sang-Won Lee

**Affiliations:** ^1^Division of Rheumatology, Department of Internal Medicine, Yonsei University College of Medicine, Seoul, South Korea; ^2^Department of Medicine, Yonsei University College of Medicine, Seoul, South Korea; ^3^Institute for Immunology and Immunological Diseases, Yonsei University College of Medicine, Seoul, South Korea; ^4^Division of Endocrinology and Metabolism, Department of Internal Medicine, Hallym University Sacred Heart Hospital, Anyang, South Korea

**Keywords:** antineutrophil cytoplasmic antibody, vasculitis, fatty liver index, mortality, cerebrovascular accident

## Abstract

**Background:**

This study investigated whether the fatty liver index (FLI) could predict all-cause mortality and cerebrovascular accident (CVA) during follow-up in patients with antineutrophil cytoplasmic antibody (ANCA)-associated vasculitis (AAV) without substantial liver disease.

**Methods:**

The medical records of 75 AAV patients with AAV were retrospectively reviewed. An equation for the FLI is as follows: FLI = (e^0.953×loge(triglycerides)+0.139×BMI+0.718×loge(GGT)+0.053×waistcircumference–15.745^)/(1 + e^0.953×loge(triglycerides)+0.139×BMI+0.718×loge(GGT)+0.053×waistcircumference–15.745^) × 100. The cut-offs of the FLI were obtained using the receiver operator characteristic (ROC) curve analysis.

**Results:**

The mean age at AAV diagnosis was 59.1 years and 42.7% were male. Eight patients (10.7%) died and 8 patients had CVA during follow-up. When the cut-offs of the FLI for all-cause mortality and CVA were set as the FLI ≥ 33.59 and the FLI ≥ 32.31, AAV patients with the FLI over each cut-off exhibited a higher risk for all-cause mortality or CVA than those without (RR 8.633 and 8.129), respectively. In addition, AAV patients with the FLI over each cut-off exhibited a significantly lower cumulative patients’ survival rate or CVA-free survival rate than those without, respectively. In the multivariable Cox analysis, only the FLI ≥ 33.59 at AAV diagnosis was an independent predictor of all-cause mortality during follow-up in AAV patients (HR 10.448).

**Conclusion:**

The FLI at AAV diagnosis can be a potential independent predictor of all-cause mortality and CVA during follow-up in AAV patients. We suggest that physicians measure the FLI at AAV diagnosis and pay more attention to those with a high FLI value for prevention of future mortality and CVA.

## Introduction

Antineutrophil cytoplasmic antibody (ANCA)-associated vasculitis (AAV) is a small vessels vasculitis and primarily affects the capillaries and adjacent venules and arterioles (1). AAV is primarily categorised into three subtypes such as microscopic polyangiitis (MPA), granulomatosis with polyangiitis (GPA), and eosinophilic granulomatosis with polyangiitis (EGPA) (2) and it is occasionally classified as myeloperoxidase (MPO)-ANCA vasculitis, proteinase 3 (PR3)-ANCA vasculitis, and ANCA-negative vasculitis (3). AAV can affect almost all organs in the body, and may inflict serious damage to the major organs, leading to death (4, 5). A recent study reported that the rate of all-cause mortality in AAV patients was 38.4 per 1,000 patient-years and the standardised mortality ratio was 2.3 (6). Another study in 2014 provided a global age-standardised mortality rate for AAV of 0.53 deaths per million inhabitants (0.62 for men and 0.46 for women) for AAV (7). We also previously reported that 1-, 5-, and 10-year cumulative patient survival rates of 96.1, 94.8, and 92.8%, respectively, in Korean patients with AAV (8). Therefore, it is believed that in addition to accurate early diagnosis and early treatment initiation, the efforts to discover new indices to predict poor outcomes, particularly all-cause mortality may improve the prognosis of AAV (9, 10).

The fatty liver index (FLI), which is calculated based on an equation composed of body mass index (BMI), waist circumference, triglyceride (TG), and gamma-glutamyl transferase (GGT), was recently introduced as a surrogate marker of non-alcoholic fatty liver disease (NAFLD), and its predictability for NAFLD was validated by imaging methods (11). NAFLD was reportedly associated with metabolic disorders including type 2 diabetes mellitus (T2DM), hypertension, cerebrovascular accident (CVA), cardiovascular disease (CVD), and chronic kidney diseases (12–15). Although this index was initially created to assess the presence and degree of hepatic steatosis in individuals, FLI recently has been also considered an emerging marker of chronic inflammation-related cardiovascular disease regarding the close association between hepatic steatosis subclinical atherosclerosis. On the other hand, to date, the predictive potential of the FLI for CVD and all-cause mortality has been reported in the various ethnic population or patients with diverse diseases (16–19). Moreover, the FLI was also reportedly associated with the risk of CVD and all-cause mortality in the general Korean population (20, 21). Therefore, it could be reasonably assumed that the FLI may predict the risk of all-cause mortality by predicting the development of various complications associated with metabolic diseases such as CVA or CVD through NAFLD-related mechanisms, induced by systemic inflammation in AAV patients. However, despite these hypotheses, no studies to date have examined whether the FLI predicts all-cause mortality or the occurrence of CVA, and CVD among AAV patients. Given the serious systemic complications and poor outcomes of AAV, it is believed that proving the clinical implications of the FLI will be of great help in selecting the best treatment option. Hence, here, we investigated whether the FLI at AAV diagnosis could predict all-cause mortality and poor outcomes of AAV during follow-up in AAV patients without substantial liver disease.

## Materials and Methods

### Study Subjects

In this study, the medical records of 75 patients with AAV were selected from the Severance Hospital ANCA-associated VasculitidEs (SHAVE) observational cohort. The patients who fulfilled the inclusion criteria were retrospectively reviewed. The inclusion criteria were (i) first classified as AAV at the Division of Rheumatology, Department of Internal Medicine, Yonsei University College of Medicine, Severance Hospital between 2000 and 2021; (ii) met both the 2012 revised Chapel Hill Consensus Conference nomenclature of vasculitis and the 2007 European Medicine Agency algorithm for AAV and polyarteritis nodosa (1, 2); (iii) availability of complete medical records, which were sufficient to obtain clinical, laboratory, radiological and histological data at diagnosis as well as information about poor outcomes of AAV including all-cause mortality, and the occurrence of CVA and CVD during follow-up; (iv) medical records that included all four variables for calculating the FLI at diagnosis; (v) follow-up periods of longer than 3 months; (vi) absence of chronic liver diseases such as B and C viral hepatitis and liver cirrhosis or structural abnormalities on liver imaging including, computed tomography or ultrasonography (22); (vii) no right–sided heart failure which may affect liver function; (viii) absence of concurrent serious medical conditions such as malignancies, infectious diseases requiring the hospitalisation, or systemic vasculitis other than AAV; and (ix) no history of exposure to immunosuppressive drugs or prednisolone > 20 mg/day before AAV diagnosis. This study was approved by the Institutional Review Board (IRB) of Severance Hospital (Seoul, South Korea, IRB No. 4-2020-1071) and conducted according to the principles of the Declaration of Helsinki. Given the retrospective design of the study and the use of anonymised patient data, the requirement for written informed consent was waived.

### Data at Diagnosis

Epidemiological, clinical, and laboratory data were collected (Table 1). AAV-related data included AAV subtypes, ANCA types and positivity, Birmingham vasculitis activity score version 3(BVAS), and five-factor score (FFS) (23, 24). Comorbidities such as T2DM and hypertension were evaluated using questionnaires on medical history including medication use. Along with laboratory results, erythrocyte sedimentation rate (ESR), C-reactive protein (CRP), liver-related variables, cholesterol profiles, and the FLI-related variables were measured from blood samples obtained after an overnight fast. Liver-related variables included prothrombin time (international normalised ratio, [INR]), alkaline phosphatase (ALP), aspartate aminotransferase (AST), alanine aminotransferase (ALT), GGT and total bilirubin.

**TABLE 1:** Baseline characteristics of AAV patients (*N* = 75).

Variables	Values
***At the time of diagnosis***	
**Demographic data**	
Age (years)	59.1 (20.8)
Male gender [N, (%)]	32 (42.7)
BMI (kg/m^2^)	22.2 (4.2)
Alcohol history [N, (%)]	
**AAV subtypes [N, (%)]**	
MPA	43 (57.3)
GPA	17 (22.7)
EGPA	15 (20.0)
**ANCA types and positivity [N, (%)]**	
MPO-ANCA (or P-ANCA) positivity	56 (74.7)
PR3-ANCA (or C-ANCA) positivity	10 (13.3)
Both ANCA positivity	3 (4.0)
ANCA negativity	12 (16.0)
**AAV-specific indices**	
BVAS	15.0 (11.0)
FFS	1.0 (1.0)
**Comorbidities [N, (%)]**	
T2DM	25 (33.3)
Hypertension	27 (36.0)
**Laboratory results**	
White blood cell count (/mm^3^)	9,060.0 (6,700.0)
Haemoglobin (g/dL)	10.1 (3.7)
Platelet count (× 1000/mm^3^)	283.0 (186.0)
Fasting glucose (mg/dL)	106.0 (38.0)
Blood urea nitrogen (mg/dL)	22.9 (29.4)
Creatinine (mg/dL)	1.2 (2.5)
Serum albumin (g/dL)	3.4 (0.8)
**Acute phase reactants**	
ESR (mm/hr)	62.0 (64.0)
CRP (mg/L)	14.1 (91.6)
**Liver-related variables**	
Prothrombin time (INR)	1.0 (0.2)
ALP (IU/L)	69.0 (38.0)
AST (IU/L)	18.0 (10.0)
ALT (IU/L)	15.0 (14.0)
Total bilirubin (mg/dL)	0.5 (0.3)
**Cholesterol profile**	
Total cholesterol (mg/dL)	163.0 (71.0)
HDL-cholesterol (mg/dL)	44.0 (32.0)
LDL-cholesterol (mg/dL)	85.5 (51.0)
**Metabolic syndrome [N, (%)]**	48 (64.0)
**FLI-related variables**	
TG (mg/dL)	126.0 (71.0)
BMI (kg/m^2^)	22.2 (4.2)
GGT (IU/L)	36.0 (64.0)
Waist circumference (cm)	85.3 (11.7)
FLI	32.9 (43.0)
***During the follow-up duration***	
**Poor outcomes [N, (%)]**	
All-cause mortality	8 (10.7)
Relapse	24 (32.0)
ESRD	20 (26.7)
CVA	8 (10.7)
CVD	9 (12.0)
**Follow-up duration based on each poor outcome (months)**	
All-cause mortality	34.1 (60.3)
Surviving patients	39.8 (60.1)
Deceased patients	6.7 (8.7)
Relapse	20.8 (43.4)
ESRD	25.3 (56.6)
CVA	32.7 (60.0)
CVD	32.9 (59.5)
**Medications [N, (%)]**	
Glucocorticoids	73 (97.3)
Cyclophosphamide	45 (60.0)
Rituximab	13 (17.3)
Mycophenolate mofetil	10 (13.3)
Azathioprine	45 (60.0)
Tacrolimus	8 (10.7)
Methotrexate	8 (10.7)
Plasma exchange	6 (8.0)

*Values are expressed as a median (interquartile range, IQR) or N (%). AAV, ANCA-associated vasculitis; ANCA, antineutrophil cytoplasmic antibody; BMI, body mass index; MPA, microscopic polyangiitis; GPA, granulomatosis with polyangiitis; EGPA, eosinophilic granulomatosis with polyangiitis; MPO, myeloperoxidase; P, perinuclear; PR3, proteinase 3; C, cytoplasmic; BVAS, Birmingham vasculitis activity score; FFS, five-factor score; T2DM, type 2 diabetes mellitus; ESR, erythrocyte sedimentation rate; CRP, C-reactive protein; ALP, alkaline phosphatase; AST, aspartate aminotransferase; ALT, alanine aminotransferase; HDL, high density lipoprotein; LDL, low density protein; FLI, fatty liver index; TG, triglyceride; GGT, gamma-glutamyl transferase; ESRD, end-stage renal disease; CVA, cerebrovascular accident; CVD, cardiovascular disease.*

### Data Collected During Follow-Up

During follow-up, poor outcomes including all-cause mortality, relapse, end-stage renal disease (ESRD), CVA, and CVD were assessed. ESRD, CVA and CVD were defined based on BVAS and FFS (23, 24). The follow-up period based on all-cause mortality was defined as the period between AAV diagnosis and the last visit for surviving patients and as the period between AAV diagnosis and death for deceased patients. For patients with a poor outcome, the follow-up period based on each poor outcome was defined as the period between AAV diagnosis and the occurrence of each poor outcome. Conversely, for patients without poor outcomes, the follow-up period was defined as the period between AAV diagnosis and the last visit. The medications that were administered were also assessed.

### Fatty Liver Index Calculation

FLI = (e^0.953×*loge(triglycerides)+0.139*×*BMI+0.718*×*loge(GGT)+0.053*×*waistcircumference*–15.745^)/(1 + e^0.953×*loge(triglycerides)+0.139*×*BMI+0.718*×*loge*(*GGT*) + 0.053×*waistcircumference*–15.745^) × 100 (11).

### Statistical Analysis

All statistical analyses were performed using IBM SPSS Statistics for Windows, version 26 (IBM Corp., Armonk, NY, United States). Continuous variables were expressed as medians with interquartile ranges, whereas categorical variables were expressed as numbers (percentages). Significant differences between the two categorical variables were analysed using the chi-square and Fisher’s exact tests. The Mann-Whitney *U* test was used to compare significant differences between two continuous variables. Significant differences among more than three continuous variables were investigated using the Kruskal-Wallis test. We conducted the ROC curve analysis with all-cause mortality as a state variable and the FLI as a test variable in the statistical analysis and obtained the sensitivity and specificity of each value of the FLI. We set the FLI of which the sum of sensitivity and the specificity was highest among these FLI values as the optimal cut-off for all-cause mortality. The relative risk (RR) of the cut-off for the high AAV activity was analysed using contingency tables and the chi-square test. The cumulative survival rates were compared between the two groups was analysed using the Kaplan-Meier survival analysis with the log-rank test. The multivariable Cox hazards model using variables with statistical significance on the univariable Cox hazard model was conducted to appropriately obtain the hazard ratios (HRs) during the considerable follow-up period. Statistical significance was set *P* < 0.05.

## Results

### Baseline Characteristics

The values for the detailed variables are summarised in Table 1. The mean age at AAV diagnosis was 59.1 years and 32 (42.7%) patients were male. Of the total, 43 patients were classified as MPA, 17 as GPA, and 15 as EGPA. Twenty-five patients (33.3%) had T2DM, while 27 patients (36.0%) had hypertension. The median prothrombin time (INR), ALP, AST, ALT, and total bilirubin were all within normal reference ranges. The median TG, BMI, GGT, waist circumference, and the FLI were 126.0 mg/dL, 22.2 kg/m^2^, 36.0 IU/L, 85.3 cm, and 32.9, respectively. During follow-up, eight patients (10.7%) died of any cause, while 24 patients experienced relapse after remission. Twenty, eight, and nine patients exhibited ESRD, CVA, and CVD, respectively. The follow-up duration based on all-cause mortality in all patients was 34.1 months. For surviving and deceased patients, they were 39.8 and 6.7 months, respectively.

### Cut-Offs of the Fatty Liver Index and Relative Risk of All-Cause Mortality and Cerebrovascular Accident

**FIGURE 1:**
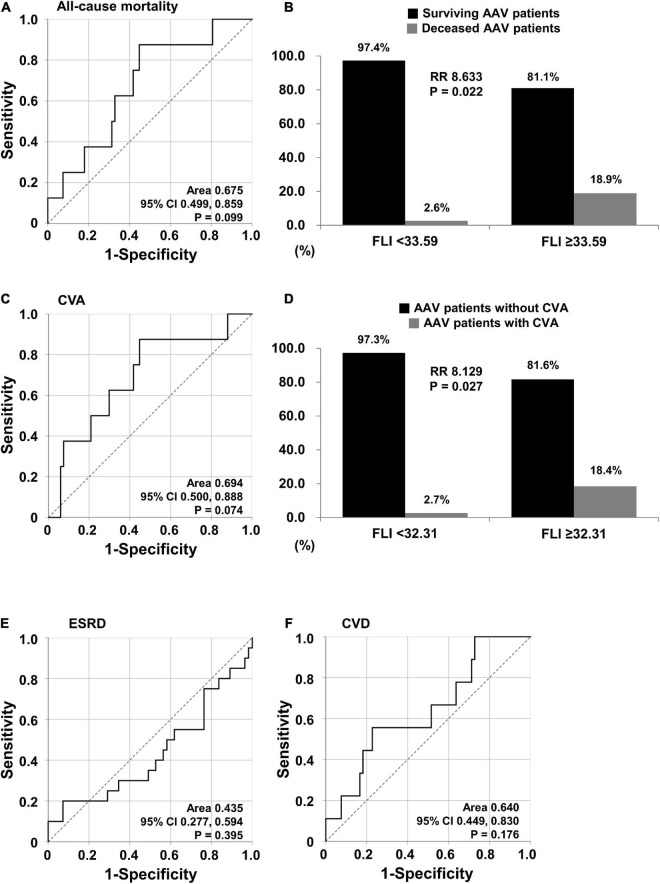
Cut-offs of the FLI for all-cause mortality and CVA. **(A)** Identification of the cut-off of the FLI for all-cause mortality using the ROC curve. **(B)** RR of all-cause mortality according to the cut-off of the FLI. **(C)** Identification of the cut-off of the FLI for CVA using the ROC curve. **(D)** RR of CVA based on the cut-off of the FLI. **(E,F)** Identification of the cut-offs of the FLI for ESRD and CVD. FLI, fatty liver index; RR, relative risk; CVA, cerebrovascular accident; ROC, receiver operator characteristic; ESRD, end-stage renal disease; CVD, cardiovascular disease.

When using the ROC curve to evaluate the predictive ability of the FLI for five poor outcomes of AAV, and with readjustment for statistical significance to *P* < 0.1 based on clinical judgment, the FLI was turned out to be associated with all-cause mortality (AUC, 0.675, 95% confidence interval CI 0.499, 0.859, *P* = 0.099) and CVA (AUC, 0.694, 95% CI 0.500, 0.888, *P* = 0.074) (Figures 1A,C). When the optimal cut-off of the FLI for all-cause mortality was set as the FLI ≥ 33.59, the sensitivity and specificity were 87.5 and 55.2%, respectively. All-cause mortality was identified more frequently in AAV patients with the FLI ≥ 33.59 than in those with the FLI < 33.59 (18.9 vs. 2.6%, *P* = 0.022). Furthermore, AAV patients with the FLI ≥ 33.59 exhibited a significantly higher risk for all-cause mortality than those with the FLI < 33.59 (RR 8.633) (Figure 1B). When the optimal cut-off of the FLI for CVA occurrence was set as the FLI ≥ 32.31, the sensitivity was 87.5% and the specificity was 53.7%. CVA occurrence was identified more frequently in AAV patients with the FLI ≥ 32.31 than those with the FLI < 33.59 (18.4 vs. 2.7%, *P* = 0.027). Furthermore, AAV patients with the FLI ≥ 32.31 exhibited a significantly higher risk for CVA occurrence than those with the FLI < 32.31 (RR 8.129) (Figure 1D). However, CVD, which was considered to be theoretically associated with NAFLD, and ESRD, which showed a significant difference (Table 2), was not predicted by the FLI (Figures 1E,F).

**TABLE 2:** Comparison of variables between surviving and deceased AAV patients.

Variables	Surviving AAV patients (*N* = 67)	Decease AAV patients (*N* = 8)	*P*-value
***At the time of diagnosis***			
**Demographic data**			
Age (years)	58.7 (19.8)	70.9 (16.7)	0.032
Male gender [N, (%)]	28 (41.8)	4 (50.0)	0.717
BMI (kg/m^2^)	22.2 (4.1)	23.1 (3.1)	0.391
Alcohol history [N, (%)]			
**AAV subtypes [N, (%)]**			0.164
MPA	36 (53.7)	7 (87.5)	
GPA	16 (23.9)	1 (12.5)	
EGPA	15 (22.4)	0 (0)	
**ANCA positivity [N, (%)]**			
MPO-ANCA (or P-ANCA) positivity	49 (73.1)	7 (87.5)	0.671
PR3-ANCA (or C-ANCA) positivity	10 (14.9)	0 (0)	0.587
Both ANCA positivity	3 (4.5)	0 (0)	1.000
ANCA negativity	11 (16.4)	1 (12.5)	1.000
**AAV-specific indices**			
BVAS	14.0 (10.0)	19.0 (9.0)	0.046
FFS	1.0 (1.0)	3.0 (1.0)	0.028
**Comorbidities [N, (%)]**			
T2DM	23 (34.3)	2 (25.0)	0.711
Hypertension	23 (34.3)	4 (75.0)	0.448
**Laboratory results**			
White blood cell count (/mm^3^)	8,645.0 (6,603.0)	11,750.0 (3,970.0)	0.286
Haemoglobin (g/dL)	10.5 (3.6)	8.3 (2.8)	0.056
Platelet count (×1,000/mm^3^)	288.0 (179.0)	271.0 (281.0)	0.844
Fasting glucose (mg/dL)	108.5 (36.0)	97.0 (63.0)	0.120
Blood urea nitrogen (mg/dL)	21.7 (29.4)	41.7 (18.0)	0.178
Creatinine (mg/dL)	1.1 (2.5)	2.5 (1.7)	0.192
Serum albumin (g/dL)	3.4 (0.8)	2.9 (1.4)	0.168
**Acute phase reactants**			
ESR (mm/hr)	62.0 (63.0)	77.0 (85.0)	0.404
CRP (mg/L)	12.2 (87.1)	77.5 (117.3)	0.363
**Liver-related variables**			
Prothrombin time (INR)	1.0 (0.2)	1.0 (0.2)	0.250
ALP (IU/L)	68.0 (37.0)	77. 0 (113.8)	0.595
AST (IU/L)	18.0 (10.0)	18.0 (18.0)	0.486
ALT (IU/L)	15.0 (14.0)	14.5 (17.8)	0.837
Total bilirubin (mg/dL)	0.4 (0.4)	0.6 (0.6)	0.063
**Cholesterol profile**			
Total cholesterol (mg/dL)	163.5 (78.0)	145.0 (86.0)	0.324
HDL-cholesterol (mg/dL)	44.0 (30.0)	26.0 (39.0)	0.065
LDL-cholesterol (mg/dL)	87.1 (54.0)	81.4 (59.0)	0.390
**Metabolic syndrome [N, (%)]**	42 (62.7)	6 (75.0)	0.703
**FLI-related variables**			
TG (mg/dL)	122.5 (71.0)	143.5 (188.0)	0.492
BMI (kg/m^2^)	22.2 (4.1)	23.1 (3.1)	0.391
GGT (IU/L)	34.0 (58.0)	123.5 (981.0)	0.135
Waist circumference (cm)	84.3 (12.3)	89.5 (10.7)	0.164
FLI	28.1 (43.5)	52.4 (54.5)	0.099
***During the follow-up duration***			
**Poor outcomes [N, (%)]**			
Relapse	19 (28.4)	5 (62.5)	0.101
ESRD	14 (20.9)	6 (75.0)	0.004
CVA	5 (7.5)	3 (37.5)	0.035
CVD	7 (10.4)	2 (25.0)	0.244
**Follow-up duration based on each poor outcome (months)**			
Relapse	24.3 (43.6)	5.2 (12.4)	0.013
ESRD	28.9 (61.7)	5.0 (26.8)	0.052
CVA	34.9 (62.9)	5.1 (10.6)	0.002
CVD	36.6 (57.5)	4.2 (7.3)	0.001
**Medications [N, (%)]**			
Glucocorticoids	65 (97.0)	8 (100)	1.000
Cyclophosphamide	40 (59.7)	5 (62.5)	1.000
Rituximab	11 (16.4)	2 (25.0)	0.621
Mycophenolate mofetil	9 (13.4)	1 (12.5)	1.000
Azathioprine	40 (59.7)	5 (62.5)	1.000
Tacrolimus	6 (9.0)	2 (25.0)	0.201
Methotrexate	8 (11.9)	0 (0)	0.588
Plasma exchange	3 (4.5)	3 (37.5)	0.014

*Values are expressed as a median (interquartile range, IQR) or N (%). AAV, ANCA-associated vasculitis; ANCA, antineutrophil cytoplasmic antibody; BMI, body mass index; MPA, microscopic polyangiitis; GPA, granulomatosis with polyangiitis; EGPA, eosinophilic granulomatosis with polyangiitis; MPO, myeloperoxidase; P, perinuclear; PR3, proteinase 3; C, cytoplasmic; BVAS, Birmingham vasculitis activity score; FFS, five-factor score; T2DM, type 2 diabetes mellitus; ESR, erythrocyte sedimentation rate; CRP, C-reactive protein; ALP, alkaline phosphatase; AST, aspartate aminotransferase; ALT, alanine aminotransferase; HDL, high density lipoprotein; LDL, low density protein; FLI, fatty liver index; TG, triglyceride; GGT, gamma-glutamyl transferase; ESRD, end-stage renal disease; CVA, cerebrovascular accident; CVD, cardiovascular disease.*

### Comparison of Variables Between Surviving and Decease Antineutrophil Cytoplasmic Antibody-Associated Vasculitis Patients

As for variables at AAV diagnosis, deceased AAV patients were older (70.9 vs. 58.7 years, *P* = 0.032) and exhibited a higher value of BVAS (19.0 vs. 14.0, *P* = 0.046) and FFS (3.0 vs. 1.0, *P* = 0.028) than surviving AAV patients. There were no statistically significant intergroup differences in liver-related variables. The FLI values were higher in deceased AAV patients than that in surviving AAV patients; however, the difference was not statistically significant (*P* = 0.099). As for variables during follow-up, deceased AAV patients had higher frequencies of ESRD (75.0 vs. 20.9%, *P* = 0.004) and CVA (37.5 vs. 7.5%, *P* = 0.035) than surviving AAV patients. However, the frequency of CVD did not differ between the two groups. Overall, the follow-up period for poor outcomes in deceased AAV patients was shorter than that in surviving AAV patients. Among the therapeutic modalities, only the number of plasma exchanges performed was significantly higher in the deceased AAV patients (Table 2).

### Comparison of Variables Between Patients With and Without Cerebrovascular Accident

As for variables at AAV diagnosis, AAV patients with CVA exhibited higher BVAS than those without CVA (18.5 vs. 14.0, *P* = 0.026), but not FFS. AAV patients with CVA exhibited the lower levels of serum albumin (2.5 vs. 3.5 g/dL, *P* = 0.004), and high density lipoprotein (HDL)-cholesterol (21.0 vs. 47.0 mg/dL), and higher levels of GGT (94.0 vs. 31.0 IU/L, *P* = 0.018), ESR (93.5 vs. 61.0 mm/hr, *P* = 0.036) and CRP (110.7 vs. 10.5 mg/L, *P* = 0.047) than in those without CVA. There were no statistically significant intergroup differences in liver-related variables. The FLI was higher in AAV patients with CVA than in those without CVA but the difference was not statistically significant (*P* = 0.074). As for variables during follow-up, AAV patients with CVA exhibited a higher frequency of all-cause mortality than those without CVA (37.5 vs. 7.5%, *P* = 0.035) (Supplementary Table 1).

### Comparison of Cumulative Survival Rates

**FIGURE 2:**
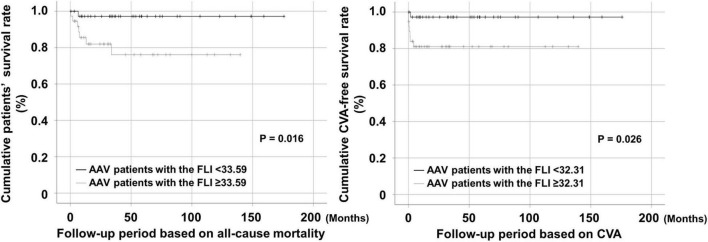
Comparison of cumulative survival rates. AAV patients with the FLI ≥ 33.59 and that ≥ 32.31 at diagnosis exhibited significantly lower cumulative patients’ and CVA-free survival rates than those without, respectively. AAV, ANCA-associated vasculitis; ANCA, antineutrophil cytoplasmic antibody; FLI, fatty liver index; CVA, cerebrovascular accident.

Thus far, the analysis may not reflect the actual clinical situation because the follow-up period was not included. Therefore, we re-evaluated the predictive ability of the FLI for all-cause mortality and CVA using Kaplan Meier survival analysis including follow-up. AAV patients with the FLI ≥ 33.59 exhibited a significantly lower cumulative patients’ survival rate than those with the FLI < 33.59 (*P* = 0.016). In addition, AAV patients with the FLI ≥ 32.31 also exhibited a significantly lower cumulative CVA-free survival rate than those with the FLI < 32.31 (*P* = 0.026) (Figure 2).

### Cox Hazards Model Analyses of Variables at Antineutrophil Cytoplasmic Antibody-Associated Vasculitis Diagnosis for All-Cause Mortality

In the univariable analysis, age (HR 1.079), FFS (HR 2.460), total bilirubin (HR 20.799), and the FLI ≥ 33.59 (HR 8.532) at AAV diagnosis were significantly associated with all-cause mortality. In the multivariable analysis, only the FLI ≥ 33.59 at AAV diagnosis was turned out to be an independent predictor of all-cause mortality during follow-up in AAV patients (HR 10.448, 95% CI 1.109, 98.398, *P* = 0.040) (Table 3).

**TABLE 3:** Cox hazards model analyses of variables at AAV diagnosis for all-cause mortality during follow-up in AAV patients.

Variables	Univariable			Multivariable		
	HR	95% CI	*P*-value	HR	95% CI	*P*-value
Age	1.079	1.001, 1.164	0.048	1.137	0.995, 1.299	0.060
Male gender	1.599	0.397, 6.437	0.509			
MPO-ANCA (or P-ANCA) positivity	2.483	0.305, 20.207	0.395			
PR3-ANCA (or C-ANCA) positivity	0.038	0.000, 142.661	0.436			
BVAS	1.100	0.985, 1.228	0.090			
FFS	2.460	1.203, 5.028	0.014	1.820	0.769, 4.310	0.173
T2DM	0.583	0.118, 2.893	0.509			
Hypertension	1.636	0.408, 6.556	0.487			
White blood cell count	1.000	1.000, 1.000	0.493			
Haemoglobin	0.699	0.463, 1.055	0.088			
Platelet count	0.999	0.993, 1.004	0.699			
Fasting glucose	0.983	0.954, 1.013	0.270			
Blood urea nitrogen	1.004	0.985, 1.023	0.689			
Creatinine	1.068	0.827, 1.379	0.615			
Serum albumin	0.386	0.116, 1.287	0.121			
ESR	1.009	0.987, 1.032	0.412			
CRP	1.006	0.995, 1.017	0.312			
Prothrombin time (INR)	14.520	0.147, 1,435.897	0.254			
ALP	1.004	0.997, 1.012	0.260			
AST	1.007	0.970, 1.046	0.706			
ALT	0.985	0.940, 1.033	0.537			
Total bilirubin	20.799	3.244, 133.354	0.001	10.249	0.316, 332.062	0.190
Total cholesterol	0.996	0.982, 1.011	0.629			
HDL-cholesterol	0.959	0.913, 1.008	0.100			
LDL-cholesterol	0.989	0.968, 1.011	0.322			
TG	1.002	0.997, 1.007	0.396			
BMI	1.087	0.879, 1.344	0.440			
GGT	1.001	1.000, 1.001	0.198			
Waist circumference	1.049	0.971, 1.133	0.222			
FLI ≥ 33.59	8.532	1.046, 69.606	0.045	10.448	1.109, 98.398	0.040

*AAV, ANCA-associated vasculitis; ANCA, antineutrophil cytoplasmic antibody; MPO, myeloperoxidase; P, perinuclear; PR3, proteinase 3; C, cytoplasmic; BVAS, Birmingham vasculitis activity score; FFS, five-factor score; T2DM, type 2 diabetes mellitus; ESR, erythrocyte sedimentation rate; CRP, C-reactive protein; ALP, alkaline phosphatase; AST, aspartate aminotransferase; ALT, alanine aminotransferase; HDL, high density lipoprotein; LDL, low density protein; BMI, body mass index; TG, triglyceride; GGT, gamma-glutamyl transferase; FLI, fatty liver index.*

## Discussion

In this study, we investigated the clinical significance of the FLI in AAV patients and obtained several interesting results. First, the FLI was associated with all-cause mortality and CVA in the ROC curve, and thus each optimal cut-off of the FLI for predicting all-cause mortality or CVA could be obtained. Second, when the cut-offs for all-cause mortality and CVA were set at 33.59 and 32.31, the RRs were 8.633 and 8.129, respectively. Third, deceased AAV patients had higher frequencies of ESRD (75.0 vs. 20.9%, *P* = 0.004) and CVA (37.5 vs. 7.5%, *P* = 0.035) than surviving AAV patients, whereas, AAV patients with CVA had a higher frequency of all-cause mortality than those without CVA (37.5 vs. 7.5%, *P* = 0.035). Fourth, AAV patients with the FLI ≥ 33.59 exhibited a significantly lower cumulative patients’ survival rate than those with the FLI < 33.59 (*P* = 0.016). In addition, AAV patients with the FLI ≥ 32.31 also exhibited a significantly lower cumulative CVA-free survival rate than those with the FLI < 32.31 (*P* = 0.026). Therefore, the FLI can predict all-cause mortality and CVA in AAV patients, suggesting the possible relationship between all-cause mortality and CVA.

**FIGURE 3:**
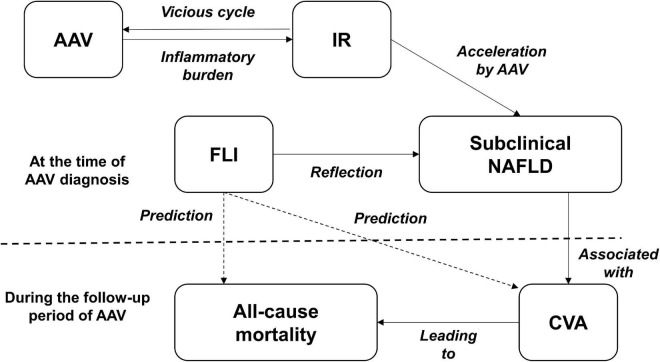
A hypothesis of the mechanism. AAV, ANCA-associated vasculitis; ANCA, antineutrophil cytoplasmic antibody; IR, insulin resistance; FLI, fatty liver index; NAFLD, non-alcoholic fatty liver disease; CVA, cerebrovascular accident.

The FLI is known to reflect the presence of NAFLD. In addition, NAFLD is reportedly closely related to current T2DM and insulin resistance (IR) and can predict the occurrence of CVA or CVD, the main causes of all-cause mortality after a certain period of time (12–15). On the other hand, liver involvement in AAV patients is very rare, and the liver-related indices are not included in the BVAS form for measuring AAV activity (23). We considered the potential FLI involvement in predicting all-cause mortality and CVA in AAV patients and hypothesised its underlying mechanism. First, we evaluated whether AAV has a direct influence on NAFLD development. Since systemic inflammation can induce IR, which in turn exacerbates systemic inflammation, a vicious cycle is formed. Moreover, such a vicious cycle between systemic inflammation and IR could, in turn, promote and accelerate the development of NAFLD (25). Furthermore, among inflammation patterns, persistent low-grade inflammation may be more closely associated with IR and NAFLD than high-grade inflammation (26) (Figure 3).

Second, we evaluated whether subclinical NAFLD is relevant to poor outcomes in patients with AAV. Although the most exact method of diagnosing NAFLD is liver biopsy, it is invasive and has limitations in actual clinical use. Ultrasonography is the most common tool used to assess the presence of NAFLD. However, it also has several limitations for the detection of milder degrees of steatosis (<33% fat in hepatocytes) (27, 28). Therefore, Bedogni et al. developed a simple scoring system called the FLI as a predictor of hepatic steatosis (11), which has been well validated in Korean populations and other ethnic groups (20, 21). The value of the FLI is expressed from 0 to 100, and when it is less than 30, NAFLD can be ruled out. The FLI ≥ 60 suggests a high likelihood of NAFLD or clinical NAFLD, whereas a value of FLI ≥ 30 but < 60 indicates an intermediate likelihood of NAFLD (29). In this study, the cut-off values of FLI were 33.59 and 32.31 for all-cause mortality and CVA, respectively. These results indicate that the presence of NAFLD might be a predictor of CVA and mortality in AAV patients, similar to the general population. Therefore, AAV patients with high FLI value need to monitor and lower its’ value by lifestyle modifications consisting of calorie restriction diet, regular exercise, and weight reduction which are traditional management of NAFLD for their prognosis.

In principle, AAV patients with abnormal liver-related variables were excluded from this study for two reasons. First, chronic liver diseases may distract the interpretation of the FLI’s ability to reflect subclinical NAFLD, which can promote progression and CVA and possible death. Second, the association between liver-related variables and AAV is weak because AAV rarely provokes progressive liver involvement or autoimmune hepatic diseases (30). A strict exclusion criteria applied in this study were as follows: ALT > 40 IU/L, AST > 40 IU/L, ALP > 115 IU/L, total bilirubin > 1.2 mg/dL, prothrombin time (INR) > 1.16, GGT > 54 IU/L, platelet count < 150,000/mm^3^, and serum albumin < 3.5 mg/dL (22). Of these variables, platelet count and serum albumin level were not included because they may be affected by the inflammatory burden in AAV patients. As GGT is one of the parameters of an equation used to calculate the FLI, it was not included in the exclusion criteria. Of the 75 patients with AAV, 11 with an ALT > 40 IU/L, one with an AST > 40 IU/L, nine with an ALP > 115 IU/L, two with a total bilirubin > 1.2 mg/dL, and seven with a prothrombin time (INR) > 1.16 were excluded. Remaining 45 AAV patients with normal liver-related variables were included and re-evaluated. The identification of the cut-offs of the FLI for predicting all-cause mortality and CVA was attempted using the ROC curve, but no cut-offs with values of *P* < 0.1 were obtained (AUC, 0.635; 95% CI, 0.286–0.984; *P* = 0.439 for all-cause mortality and AUC, 0.779; 95% CI, 0.636–0.922; *P* = 0.186 for CVA) (Supplementary Figure 1).

Alanine aminotransferase is a parameter that is closely related to liver damage. Thus, Of the 75 AAV patients, 11 patients with an ALT > 40 IU/L were excluded. With remaining 64 AAV patients, the efficacy of the FLI was re-evaluated. First, the ROC curve was used to obtain the FLI cut-off value for predicting all-cause mortality (re-adjusted statistical significance to *P* < 0.1). The FLI was associated with all-cause mortality (AUC, 0.714; 95% CI, 0.539–0.890; *P* = 0.051). When the optimal cut-off of FLI for all-cause mortality was set as ≥ 33.59, the sensitivity and specificity were 87.5 and 60.7%, respectively. All-cause mortality was identified more frequently in AAV patients with the FLI ≥ 33.59 than in those with the FLI < 33.59 (24.1 vs. 2.9%; *P* = 0.019). Furthermore, AAV patients with the FLI ≥ 33.59 exhibited a significantly higher risk for all-cause mortality than those with the FLI < 33.59 (RR, 10.818) (Supplementary Figure 2). However, significant cut-offs of the FLI for predicting CVA (*P* = 0.105) and CVD (*P* = 0.180) were not obtained using the ROC curve analysis (Supplementary Figure 3). AAV patients with the FLI ≥ 33.59 exhibited a significantly lower cumulative patients’ survival rate than those with the FLI < 33.59 (*P* = 0.007) (Supplementary Figure 4). Therefore, the FLI reflecting NAFLD at the AAV diagnosis could predict all-cause mortality during follow-up in AAV patients regardless of ALT levels. However, the FLI at diagnosis could predict the occurrence of CVA in all AAV patients but not in those with normal ALT levels.

For variables other than ALT which is closely related to liver damage, the systemic inflammation due to AAV could subsequently increase their serum level and should be considered when analysing the result. As for AST, FIB-5, for which AST is a parameter located in the numerator of the equation, was proven to significantly predict ESRD in patients with MPA and GPA without substantial liver diseases (31). In addition, AST levels may be elevated in cardiovascular diseases including myocarditis and ischaemic heart disease in AAV patients (32). ALP reportedly predicts CVD occurrence and serious complications of chronic kidney disease (33). Total bilirubin is reported to have an anti-inflammatory mechanism and can be elevated to subside inflammation (34). In addition, prothrombin time is considered to be linked to inflammatory indices (35). Meanwhile, ALT is strongly associated with NAFLD, and its elevation is largely attributed to NAFLD severity (36).

### Advantages

The primary advantage of this study was that we demonstrated for the first time that the FLI could predict all-cause mortality in patients with AAV. We could generate a hypothesis that NAFLD occurrence may be increased through the axis of systemic inflammation and IR in patients with AAV, which does not directly affect liver. Also, instead of providing the absolute FLI cut-off value, we provided a method for obtaining the cut-off value of the FLI at diagnosis for predicting all-cause mortality during follow-up in AAV patients, which may enable the application of this concept to AAV patients with different ethnic and geographical backgrounds. In particular, despite the small number of study subjects, our study has additional three advantages: First, the SHAVE cohort from which the study subjects were collected is the cohort containing the largest number of AAV patients in Korea. Second, the same three rheumatologists made new diagnoses of AAV or participated in the reclassification of AAV in existing patients. Third, since the same three rheumatologists applied the same classification criteria for AAV, the diagnosis concordance rate was high.

### Limitations

This study has several limitations. Because GGT is not necessarily included in routine tests for AAV diagnosis, the number of AAV patients with GGT results included in this study was insufficient to clarify whether our results could be applied to all patients with AAV. In addition, this study did not reflect the dynamic change through the evaluation of NAFLD at every visit because of the limitations of the retrospective study design. In addition, liver biopsy and measurements of biomarkers related to liver fibrosis or steatohepatitis were not performed to evaluate the exact degree of NAFLD, that is, the severity of liver fibrosis or steatohepatitis. Therefore, we could not determine the association between severe form of NAFLD and health outcome in our patients. Although glucocorticoids and immunosuppressive drugs could be controlled through the drug utilisation review system, we could not strictly control the drug-related occurrence of hepatitis or NAFLD because we did not obtain complete information about general or herbal medications. On the other hand, if we showed the status of the circulating inflammatory load including hs-CRP and fibrinogen, we could have more concretely proved the hypothesis that inflammatory burden might induce IR and accelerate the occurrence of subclinical NAFLD which is associated with all-cause mortality. However, because this study was conducted retrospectively, we could not demonstrate it directly. Future prospective studies with larger numbers of AAV patients that include transient elastography along with FLI assessments will solve these limitations, validate our results, and will further suggest the clinical implications of the FLI in AAV patients.

## Conclusion

The FLI, a validated surrogate marker of NAFLD, at AAV diagnosis can be a potential independent predictor of all-cause mortality and the occurrence of CVA during follow-up in patients with AAV. This finding suggests that the presence of NAFLD may contribute to poor outcomes in these patients. Moreover, we demonstrated that FLI is a simple and useful marker for the classification of prognosis in patients with AAV. Given the higher prevalence of all-cause mortality and CVA in AAV patients, we suggest that physicians measure the FLI when AAV is diagnosed and pay more attention to those with NAFLD or a high FLI value for prevention of future mortality and CVA.

## Data Availability Statement

The raw data supporting the conclusions of this article will be made available by the authors, without undue reservation.

## Ethics Statement

The studies involving human participants were reviewed and approved by the Institutional Review Board (IRB) of Severance Hospital (Seoul, South Korea, IRB No. 4-2020-1071). Written informed consent for participation was not required for this study in accordance with the national legislation and the institutional requirements.

## Author Contributions

PP and HC carried out the statistical analysis. PP, JH, and S-WL wrote the first draft of the manuscript. JP, SA, HC, JS, and Y-BP collected the data. JH and S-WL responsible for the conception, funding, design of the study, the guarantors of this work and, as such, had full access to all the data in the study and take responsibility for the integrity of the data and the accuracy of the data analysis. All authors corrected and approved the revisions and final version of the manuscript, contributed to the article, and approved the submitted version.

## Conflict of Interest

The authors declare that the research was conducted in the absence of any commercial or financial relationships that could be construed as a potential conflict of interest.

## Publisher’s Note

All claims expressed in this article are solely those of the authors and do not necessarily represent those of their affiliated organizations, or those of the publisher, the editors and the reviewers. Any product that may be evaluated in this article, or claim that may be made by its manufacturer, is not guaranteed or endorsed by the publisher.
