# Supplementary material for: Fatty Liver Index Independently Predicts All-Cause Mortality in Patients With Antineutrophil Cytoplasmic Antibody-Associated Vasculitis but No Substantial Liver Disease
Source: Front Cardiovasc Med. 2022 Jun 23;9:848121. doi: 10.3389/fcvm.2022.848121 (PMC9259888; doi:10.3389/fcvm.2022.848121)
Supplement: Supplementary file 1 [file Table_1.DOCX]

Supplementary Material

# Supplementary Data

Supplementary Material should be uploaded separately on submission. Please include any supplementary data, figures and/or tables. All supplementary files are deposited to FigShare for permanent storage and receive a DOI.

Supplementary material is not typeset so please ensure that all information is clearly presented, the appropriate caption is included in the file and not in the manuscript, and that the style conforms to the rest of the article. To avoid discrepancies between the published article and the supplementary material, please do not add the title, author list, affiliations or correspondence in the supplementary files.

# Supplementary Figures and Tables

For more information on Supplementary Material and for details on the different file types accepted, please see [here](http://home.frontiersin.org/about/author-guidelines#SupplementaryMaterial). Figures, tables, and images will be published under a Creative Commons CC-BY licence and permission must be obtained for use of copyrighted material from other sources (including re-published/adapted/modified/partial figures and images from the internet). It is the responsibility of the authors to acquire the licenses, to follow any citation instructions requested by third-party rights holders, and cover any supplementary charges.

## Supplementary Figures


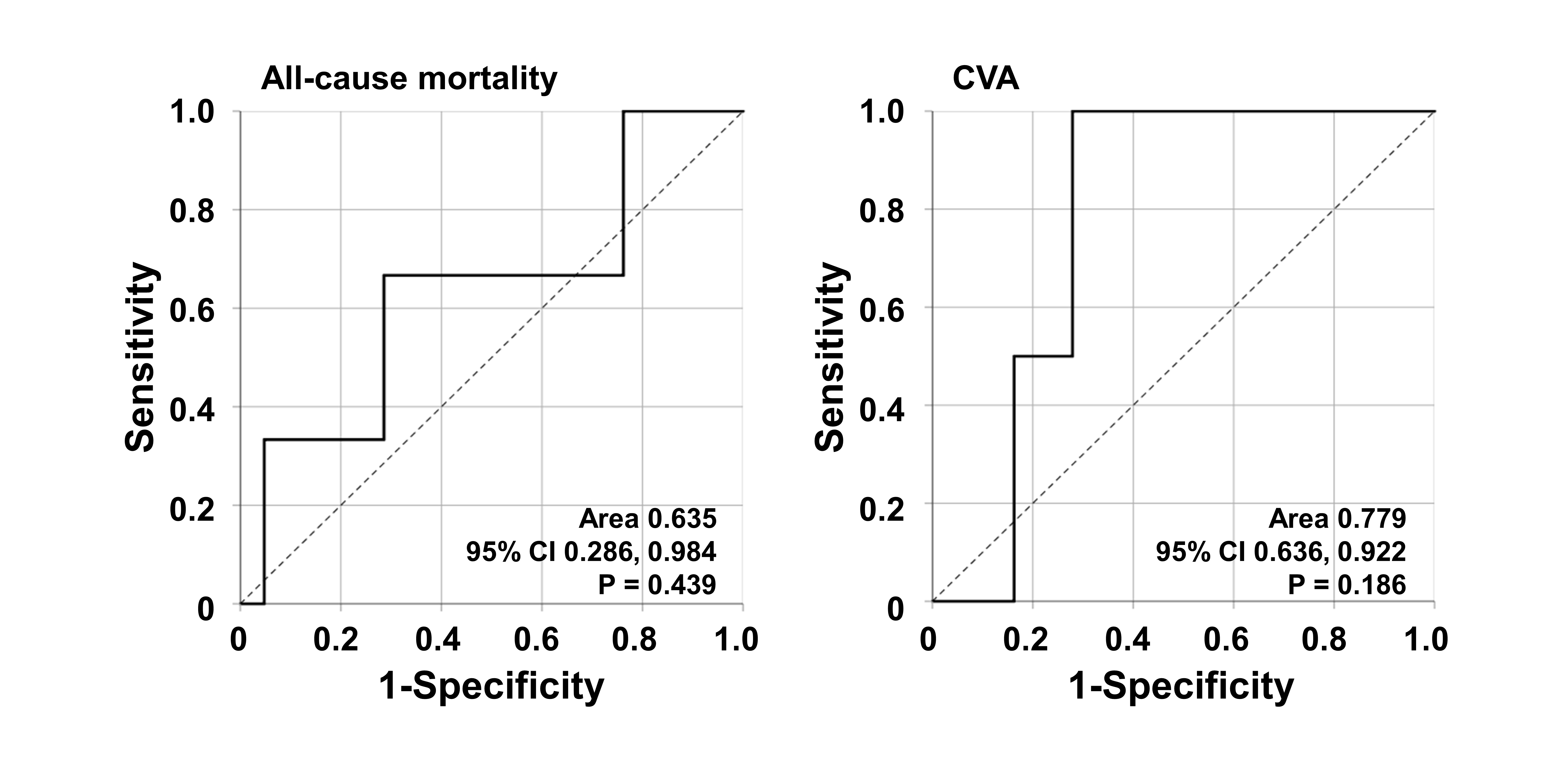


**Supplementary Figure 1.** Cut-offs of the FLI for all-cause mortality and CVA in 45 AAV patients with normal liver-related variables. No significant cut-offs of the FLI for all-cause mortality and CVA in AAV patients with normal liver-related results were obtained in the ROC curve analysis. FLI: fatty liver index; CVA: cerebrovascular accident; AAV: ANCA-associated vasculitis; ANCA: antineutrophil cytoplasmic antibody.


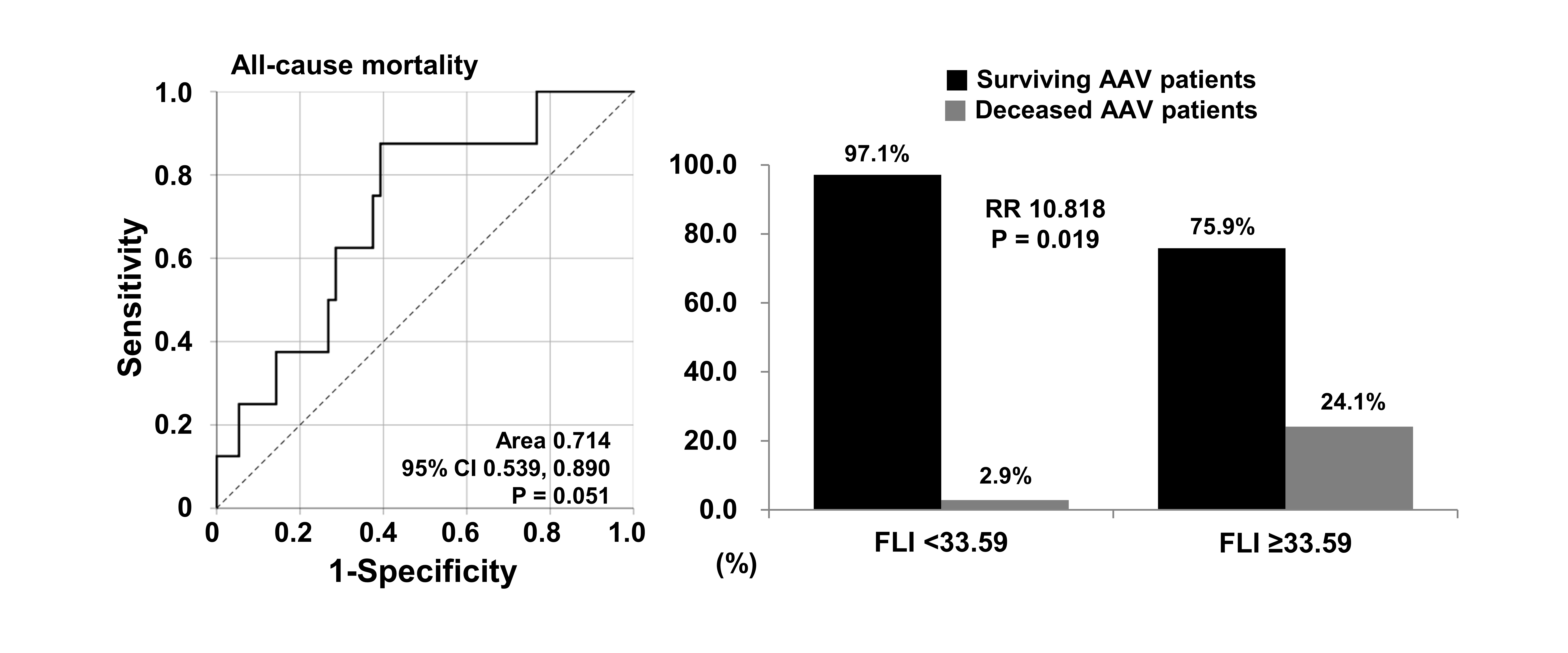


**Supplementary Figure 2.** Cut-offs of the FLI and relative risk for all-cause mortality in 64 AAV patients with normal ALT level. When the optimal cut-off of FLI for all-cause mortality was set as ≥33.59, among AAV patients with normal ALT level, AAV patients with the FLI ≥33.59 exhibited a significantly higher risk for all-cause mortality than those with the FLI <33.59. FLI: fatty liver index; AAV: ANCA-associated vasculitis; ANCA: antineutrophil cytoplasmic antibody; ALT: alanine aminotransferase.


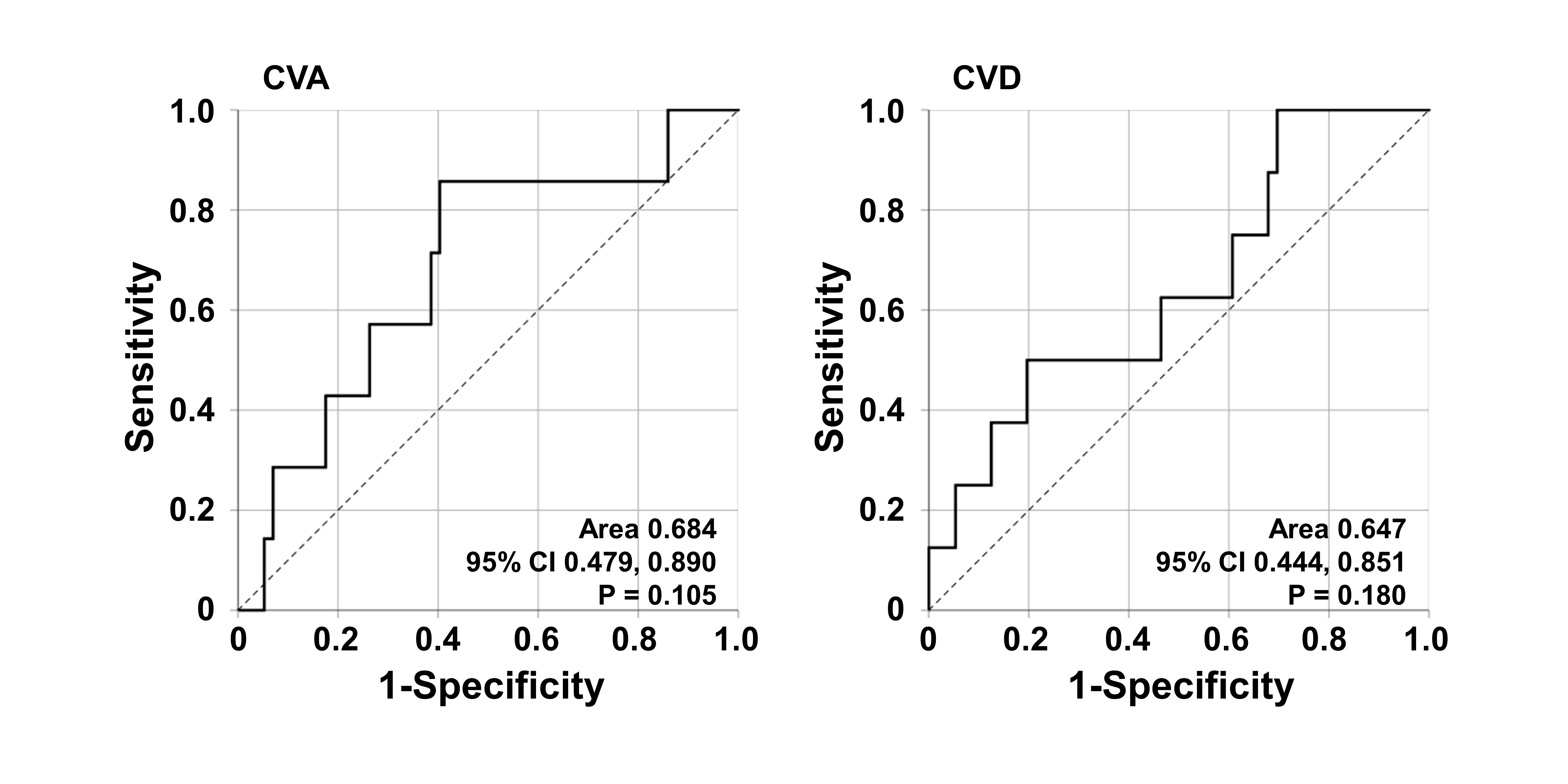


**Supplementary Figure 3.** No significant cut-offs of the FLI for CVA and CVD in 64 AAV patients with normal ALT level. No significant cut-offs of the FLI for predicting CVA (P = 0.105) and CVD (P = 0.180) were not obtained using the ROC curve analysis. FLI: fatty liver index; CVA: cerebrovascular accident; CVD: cardiovascular disease; AAV: ANCA-associated vasculitis; ANCA: antineutrophil cytoplasmic antibody; ALT: alanine aminotransferase; ROC: receiver operator characteristic.


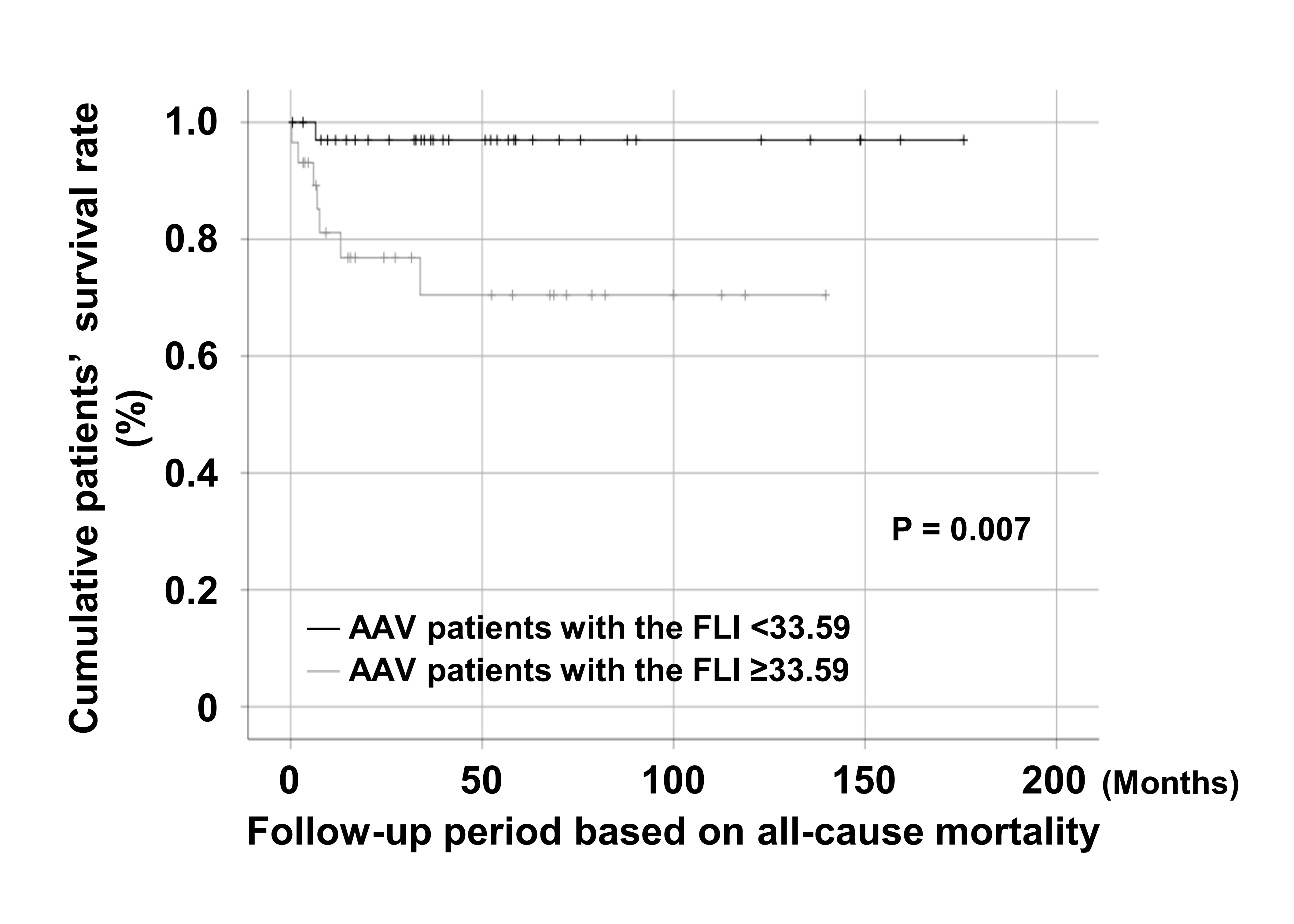


**Supplementary Figure 4.** Comparison of cumulative patients’ survival rates in 64 AAV patients with normal ALT level. AAV patients with the FLI ≥33.59 exhibited a significantly lower cumulative patients’ survival rate than those with the FLI <33.59. AAV: ANCA-associated vasculitis; ANCA: antineutrophil cytoplasmic antibody; ALT: alanine aminotransferase.

**Supplementary Table 1 Comparison of variables between AAV patients with CVA and those without CVA**

| **Variables** | **AAV patients without CVA**  **(N=67)** | **AAV patients with CVA**  **(N=8)** | **P-value** |
| --- | --- | --- | --- |
| ***At the time of diagnosis*** |  |  |  |
| **Demographic data** |  |  |  |
| Age (years) | 58.7 (20.1) | 72.0 (18.6) | 0.055 |
| Male gender (N, (%)) | 29 (43.3) | 3 (37.5) | 1.000 |
| BMI (kg/m^2^) | 22.1 (4.1) | 23.6 (4.3) | 0.186 |
| **AAV subtypes (N, (%))** |  |  | 0.317 |
| MPA | 37 (55.2) | 6 (75.0) |  |
| GPA | 15 (22.4) | 2 (25.0) |  |
| EGPA | 15 (22.4) | 0 (0) |  |
| **ANCA positivity (N, (%))** |  |  |  |
| MPO-ANCA (or P-ANCA) positivity | 50 (74.6) | 6 (75.0) | 1.000 |
| PR3-ANCA (or C-ANCA) positivity | 9 (13.4) | 1 (12.5) | 1.000 |
| Both ANCA positivity | 3 (4.5) | 0 (0) | 1.000 |
| ANCA negativity | 11 (16.4) | 1 (12.5) | 1.000 |
| **AAV-specific indices** |  |  |  |
| BVAS | 14.0 (10.0) | 18.5 (8.0) | 0.026 |
| FFS | 1.0 (1.0) | 2.0 (1.0) | 0.116 |
| **Comorbidities (N, (%))** |  |  |  |
| T2DM | 23 (34.3) | 2 (25.0) | 0.711 |
| Hypertension | 24 (35.8) | 3 (37.5) | 1.000 |
| **Laboratory results** |  |  |  |
| White blood cell count (/mm^3^) | 8,640.0 (6,340.0) | 12,250.0 (5,093.0) | 0.056 |
| Haemoglobin (g/dL) | 10.5 (3.7) | 9.0 (3.3) | 0.155 |
| Platelet count (× 1000/mm^3^) | 271.0 (167.0) | 448.0 (272.0) | 0.150 |
| Fasting glucose (mg/dL) | 106.0 (36.0) | 105.5 (81.0) | 0.817 |
| Blood urea nitrogen (mg/dL) | 22.8 (28.4) | 42.3 (29.5) | 0.280 |
| Creatinine (mg/dL) | 1.2 (2.5) | 1.6 (2.4) | 0.341 |
| Serum albumin (g/dL) | 3.4 (0.8) | 2.5 (1.0) | 0.004 |
| **Acute phase reactants** |  |  |  |
| ESR (mm/hr) | 61.0 (62.0) | 93.5 (50.0) | 0.036 |
| CRP (mg/L) | 10.5 (72.3) | 110.7 (99.78) | 0.047 |
| **Liver-related variables** |  |  |  |
| Prothrombin time (INR) | 1.0 (0.2) | 1.1 (0.2) | 0.066 |
| ALP (IU/L) | 68.0 (33.0) | 119.5 (176.3) | 0.064 |
| AST (IU/L) | 18.0 (10.0) | 19.5 (18.0) | 0.399 |
| ALT (IU/L) | 15.0 (14.0) | 17.5 (20.5) | 0.486 |
| Total bilirubin (mg/dL) | 0.5 (0.3) | 0.5 (0.3) | 0.993 |
| **Cholesterol profile** |  |  |  |
| Total cholesterol (mg/dL) | 171.0 (75.0) | 119.0 (55.0) | 0.063 |
| HDL-cholesterol (mg/dL) | 47.0 (30.0) | 21.0 (21.0) | <0.001 |
| LDL-cholesterol (mg/dL) | 86.5 (53.0) | 59.2 (50.0) | 0.232 |
| **Metabolic syndrome (N, (%))** | 41 (61.2) | 7 (87.5) | 0.245 |
| **FLI-related variables** |  |  |  |
| TG (mg/dL) | 126.0 (80.0) | 107.0 (50.0) | 0.718 |
| BMI (kg/m^2^) | 22.1 (4.1) | 23.6 (4.3) | 0.186 |
| GGT (IU/L) | 31.0 (58.0) | 94.0 (701.0) | 0.018 |
| Waist circumference (cm) | 84.7 (11.9) | 89.7 (16.4) | 0.175 |
| FLI | 31.2 (43.1) | 57.0 (55.5) | 0.074 |
| ***During the follow-up duration*** |  |  |  |
| **Poor outcomes (N, (%))** |  |  |  |
| All-cause mortality | 5 (7.5) | 3 (37.5) | 0.035 |
| Relapse | 21 (31.3) | 3 (37.5) | 0.705 |
| ESRD | 16 (23.9) | 4 (50.0) | 0.198 |
| CVD | 9 (13.4) | 0 (0) | 0.585 |
| **Follow-up duration based**  **on each poor outcome (months)** |  |  |  |
| All-cause mortality | 34.9 (60.6) | 18.3 (57.3) | 0.257 |
| Relapse | 20.8 (175.5) | 18.0 (27.2) | 0.757 |
| ESRD | 25.7 (56.6) | 8.2 (61.2) | 0.589 |
| CVD | 55.8 (59.1) | 18.3 (57.3) | 0.471 |
| **Medications (N, (%))** |  |  |  |
| Glucocorticoids | 65 (97.0) | 8 (100) | 1.000 |
| Cyclophosphamide | 41 (61.2) | 4 (50.0) | 0.706 |
| Rituximab | 12 (17.9) | 1 (12.5) | 1.000 |
| Mycophenolate mofetil | 10 (14.9) | 0 (0) | 0.587 |
| Azathioprine | 40 (59.7) | 5 (62.5) | 1.000 |
| Tacrolimus | 8 (11.9) | 0 (0) | 0.588 |
| Methotrexate | 8 (11.9) | 0 (0) | 0.588 |
| Plasma exchange | 5 (7.5) | 1 (12.5) | 0.504 |

Values are expressed as a median (interquartile range, IQR) or N (%).

AAV: ANCA-associated vasculitis; ANCA: antineutrophil cytoplasmic antibody; CVA: cerebrovascular accident; BMI: body mass index; MPA: microscopic polyangiitis; GPA: granulomatosis with polyangiitis; EGPA: eosinophilic granulomatosis with polyangiitis; MPO: myeloperoxidase; P: perinuclear; PR3: proteinase 3; C: cytoplasmic; BVAS: Birmingham vasculitis activity score; FFS: five-factor score; T2DM: type 2 diabetes mellitus; ESR: erythrocyte sedimentation rate; CRP: C-reactive protein; ALP: alkaline phosphatase; AST: aspartate aminotransferase; ALT: alanine aminotransferase; HDL: high density lipoprotein; LDL: low density protein; FLI: fatty liver index; TG: triglyceride; GGT: gamma-glutamyl transferase; ESRD: end-stage renal disease; CVD: cardiovascular disease.
